# The fatty infiltration into cervical paraspinal muscle as a predictor of postoperative outcomes: A controlled study based on hybrid surgery

**DOI:** 10.3389/fendo.2023.1128810

**Published:** 2023-02-09

**Authors:** Junbo He, Tingkui Wu, Chen Ding, Beiyu Wang, Ying Hong, Hao Liu

**Affiliations:** Department of Orthopedic Surgery, Orthopedic Research Institute, West China Hospital, Sichuan University, Chengdu, Sichuan, China

**Keywords:** cervical paraspinal muscle, fatty infiltration, cross-sectional area, hybrid surgery, cervical disc degenerative disease

## Abstract

**Background:**

The cervical paraspinal muscle (CPM) has an essential role in positioning, stabilizing and directing the cervical spine. However, information is lacking regarding the influence of CPM on outcomes following anterior cervical surgery. This study aims to evaluate the association of fatty infiltration (FI) of CPM with postoperative outcomes in patients undergoing hybrid surgery (HS) and analyze the relationship between FI and cross-sectional area (CSA) of CPM.

**Methods:**

A retrospective analysis was performed on 110 consecutive patients undergoing continuous 2-level HS. According to Goutallier classification of multifidus FI, the patients were divided into normal, moderate, and severe groups. Image J software was employed to outline and analyze CPM. Clinical outcomes and radiographic parameters were collected and evaluated for relevant comparisons.

**Results:**

Visible FI was identified in 69.1% of patients (76/110), with a propensity in elderly patients (p = 0.053). No statistically significant differences were presented among the three groups regarding pre- and postoperative clinical evaluation scores. The cervical lordosis was significantly higher in the normal group before surgery (p = 0.029). Likewise, the sagittal vertical axis (SVA) was significantly higher in the severe group than the normal group at the final follow-up (p = 0.046). The function spine unit angle and disc angle of arthroplasty levels were significantly lower in the severe group than the normal group at follow-ups. Moreover, after correction according to vertebral body area, no statistically significant relationship existed between CSA ratio and FI grade.

**Conclusion:**

CPM degeneration is common and age-related in patients with cervical disc degenerative disease. More importantly, there was a significant positive correlation between severe FI of CPM and postoperative sagittal balance disorder, particularly in C2-7 SVA and segmental alignment of arthroplasty level. Meanwhile, FI of CPM appears to have no impact on clinical outcomes and reveals small correlations to CSA.

## Introduction

The cervical paraspinal muscle (CPM) is critical for cervical spine alignment, stabilization, and direction (1). It has been estimated that neck muscles provide approximately 80% of total neck stability (2). Cross-sectional area (CSA) and fatty infiltration (FI) grade are two standard parameters used to evaluate paraspinal muscle (3–5). Preliminary research indicates that CSA of paraspinal muscles decreases with age, whereas FI grade increases, especially among the elderly (4, 5). More importantly, the consequent decrease in muscle strength results in spinal disorders. In the cervical spine, previous studies demonstrated that CPM structural and functional alterations were linked to sagittal balance disorder, poor spinal mobility, and chronic neck pain (6–9). Similarly, evidence from patients with whiplash-associated disorders revealed a significant association between cervical muscle FI and neck disability (10). Furthermore, degeneration of the vertebrae, discs, joints, and muscles around the spine may lead to spinal degeneration. Several studies have established that muscle weakness-related spinal instability may contribute to cervical disc degenerative disease (CDDD) (2, 11, 12). Therefore, insights into neck muscle condition *in vivo* are important for comprehending and minimizing risks of cervical spine disorders.

However, although the deleterious effect of paraspinal muscle in patients undergoing spine surgery is well documented, there has been limited data examining how CPM status impacts clinical and radiographical outcomes after cervical spine surgery (13). Furthermore, reports on the correlation between CPM and the outcomes of anterior surgery are even rarer than those of the posterior surgery with direct muscle invasion. Yet the utilization of former in CDDD is as high as 80% (14). In terms of clinical results, a recent retrospective cohort study revealed a significant relevance between worsening cervical paraspinal degeneration and patient-reported outcomes after anterior cervical discectomy and fusion (ACDF) (15) and recommended the proven qualitative assessment of FI during preoperative evaluation (16). However, that study included only the absolute value of muscle area. On the other hand, as reported by Thakar et al, the muscle-vertebral body area ratios were more helpful in eliminating biases arising from variations in patient build (17). In their retrospective study, greater segmental kyphotic change after central corpectomy was proved to correlate with worse CSA of cervical musculature. Nonetheless, the influence of different surgical approaches on cervical sagittal balance remained debatable, especially in CDA where the immediate stability may be less but the mobility is higher. Previous studies illustrated that the fusion implant could reinstate cervical lordosis (18), and the overall cervical alignment after CDA tends to lose lordosis (19). Moreover, the incident rate of segmental kyphosis was relatively higher after CDA (20). Thus, compared with fusion surgery, replacement prosthesis may be affected diversely by different statuses of CPM. The hybrid surgery (HS) with an *in vivo* contrast condition was therefore included in this study.

We hypothesized that fusion and replacement prostheses in HS might behave differently, and that this behavior would be influenced by the degree of fatty infiltration of the CPM. To the best of our knowledge, this is the first study to evaluate the impact of FI grade of CPM on outcomes following HS and its relationship with preoperative CSA.

## Methods

### Study design and patient selection

This was a retrospective and comparative study of patients undergoing two-level HS in our hospital from January 2011 to July 2020. Informed consent was obtained from all patients, and the study protocol was approved by the Ethics Committee of our institution (Project License number: 20190946). The eligibility criteria required a diagnosis of refractory CDDD with symptomatic radiculopathy and/or myelopathy at two contiguous levels from C-3 to C-7 confirmed by preoperative radiographical findings. Exclusion criteria included any prior spine surgery at operative levels, severe facet arthritis, fracture, infection, tumor, and severe osteoporosis (T-score ≤ − 2.5). The implant was selected based on preoperative segmental features. In this regard, CDA was performed at a mobile and soft-herniation segment. ACDF was performed in the case of segmental instability (sagittal plane translation >3.5 mm or angular motion >20°), angular motion <2°, a disc height loss of >50%, or severe facet joint degeneration. In all cases, a Prestige-LP prosthesis (Medtronic Sofamor Danek, Memphis, TN) was inserted into a well-prepared arthroplasty level, and a Zero-P implant (Synthes, Oberdorf, Switzerland) packed with β-tricalcium phosphate was used as a stand-alone arthrodesis implant. The operations were conducted *via* an anterior right-sided approach by the same senior spine surgeon.

### Outcome assessment

**Figure 1:**
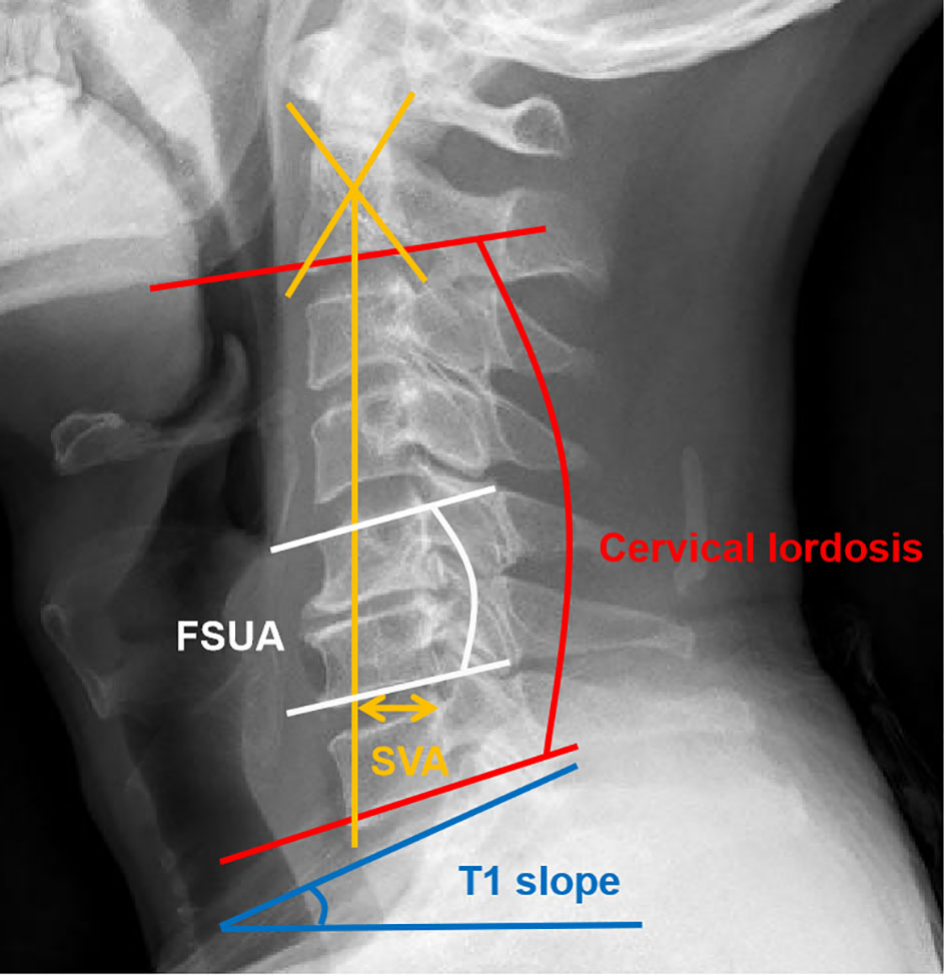
Measurements of the cervical sagittal alignment parameters. (1) Red line: cervical lordosis (CL); (2) yellow line: sagittal vertical axis (SVA); (3) white line: functional spine unit angle (FSUA); (4) blue line: T1 slope (TS).

Clinical outcomes were evaluated preoperatively and postoperatively at a minimum 1-year follow-up and compared, including Japanese Orthopaedic Association (JOA) score, neck disability index (NDI), and visual analog scale (VAS). Radiographical outcomes were assessed *via* pre- and postoperative anteroposterior, lateral, and flexion-extension radiographs (**Figure 1**). The following parameters were evaluated: (1) cervical lordosis (CL), defined as the angle between the inferior end plate of C2 and the inferior end plate of C7; (2) sagittal vertical axis (SVA), defined as the distance from the posterior, superior corner of C7 to the plumbline from the center of C2; and (3) T1 slope (TS), defined as the angle between a horizontal line and the superior endplate of T1. If TS is invisible due to anatomical interference, C7 slope was converted to T1 slope using the formula: T1 slope = (C7 slope + 0.54)/0.88 (21). Furthermore, the Cobb method was used to determine the functional spine unit angle (FSUA), arthroplasty disc angle (ADA), range of motion (ROM) of C2-C7, and ROM of arthroplasty segment (22).

### Muscle evaluations

**Figure 2:**
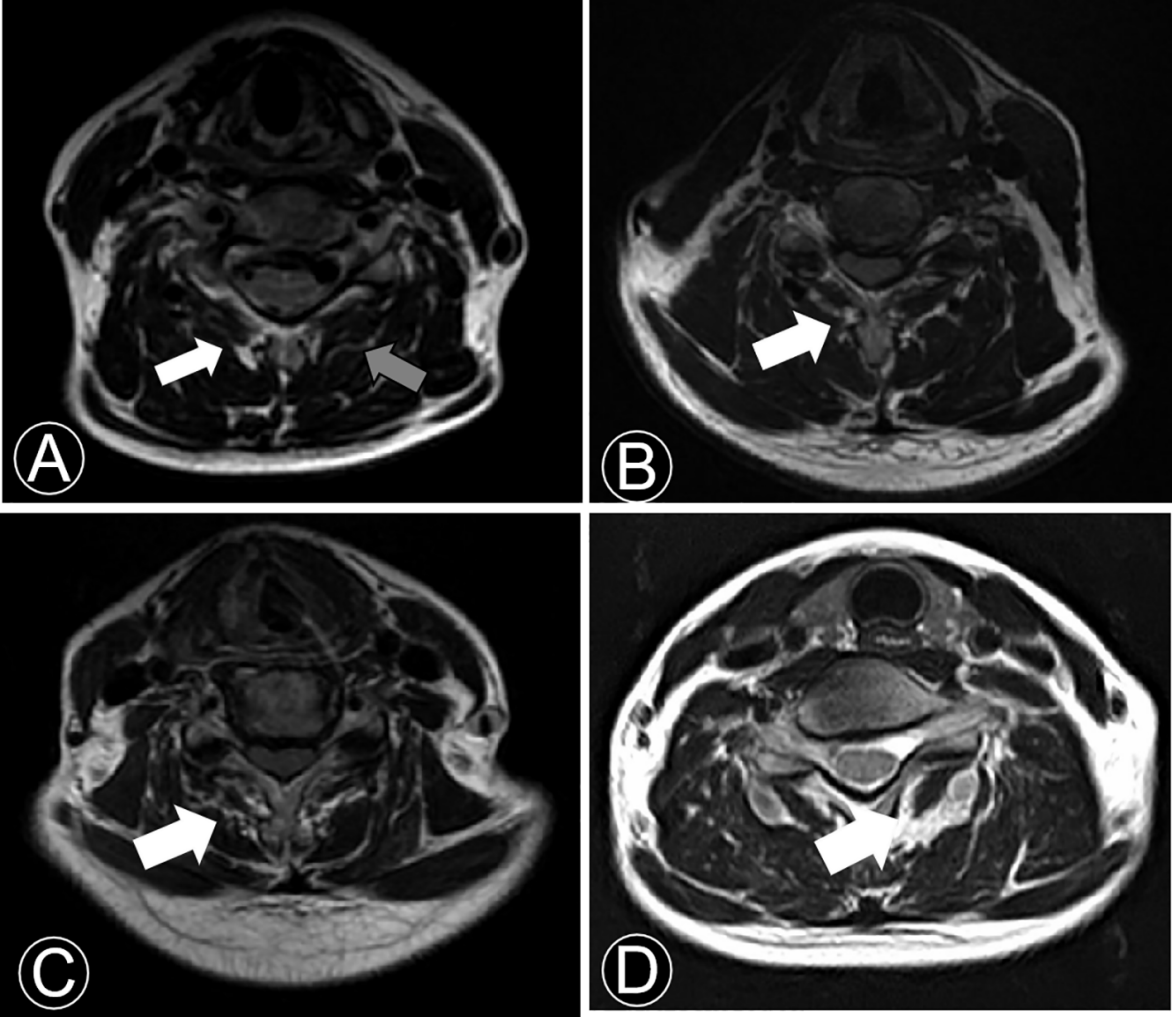
T2-weighted axial MRI section demonstrating fatty infiltration of muscle multifidus belly at C5/6. **(A)** Goutalier Grade 0 (grey arrow), Goutalier Grade 1 (white arrow); **(B)** Goutalier Grade 2. **(C)** Goutalier Grade 3. **(D)** Goutalier Grade 4.

Qualitative and quantitative evaluations of CPM were preoperatively conducted. All measurements were conducted on an axial T2 weight section from MRI. This study included the degree of fat infiltration at the C5/6 level as a representative, which is a common practice in CPM studies (7, 10, 15, 16). Goutallier classification of paracervical muscle was graded on a 0-4 scale based on a qualitative assessment of fat atrophy of muscle multifidus belly (**Table 1** **;** **Figure 2**) (15, 23). Goutalier 0, 1, 2, 3, and 4 were defined as having no visible fat streaks in the muscle, minimal fatty streaks in the muscle, fat evident but more muscle present, equal quantities of fat and muscle, and more fat have shown, respectively.

**Table 1:** Demographic and perioperative features.

Variables	Normal	Moderate	Severe	P Value
Goutalier grade	0-1	1.5-2	2.5-4	
Number of patients, n	34	38	38	
Age, years †	47.0 ± 7.7	48.4 ± 7.2	50.9 ± 6.8	0.053
Gender (female), n (%) §	18 (52.9%)	25 (65.8%)	29 (76.3%)	0.114
BMI, kg/m^2^ ‡	23.9 ± 2.9	24.4 ± 2.8	23.7 ± 2.6	0.539
ALP †	67.6 ± 21.1	65.5 ± 16.0	71.4 ± 19.7	0.324
Arthroplasty segment ¶				0.641
C3/4	1	1	1	
C4/5	11	18	19	
C5/6	8	10	9	
C6/7	14	9	9	
Fusion location §				0.387
Up	17	15	13	
Down	17	23	25	
Operation time (minutes) †	137.2 ± 28.4	133.1 ± 23.3	143.6 ± 37.6	0.241
Blood loss (ml) †	78.2 ± 50.1	70.0 ± 43.9	73.7 ± 67.8	0.340
Follow-up time (months) ‡	22.5 ± 16.3	21.9 ± 15.7	25.7 ± 14.1	0.522

BMI, body mass index; BMD, bone mineral density; ALP, alkaline phosphatase.

† Kruskal-Wallis H-test.

‡ One-way analysis of variance.

§ Chi-square test.

¶Fisher exact test.

**Figure 3:**
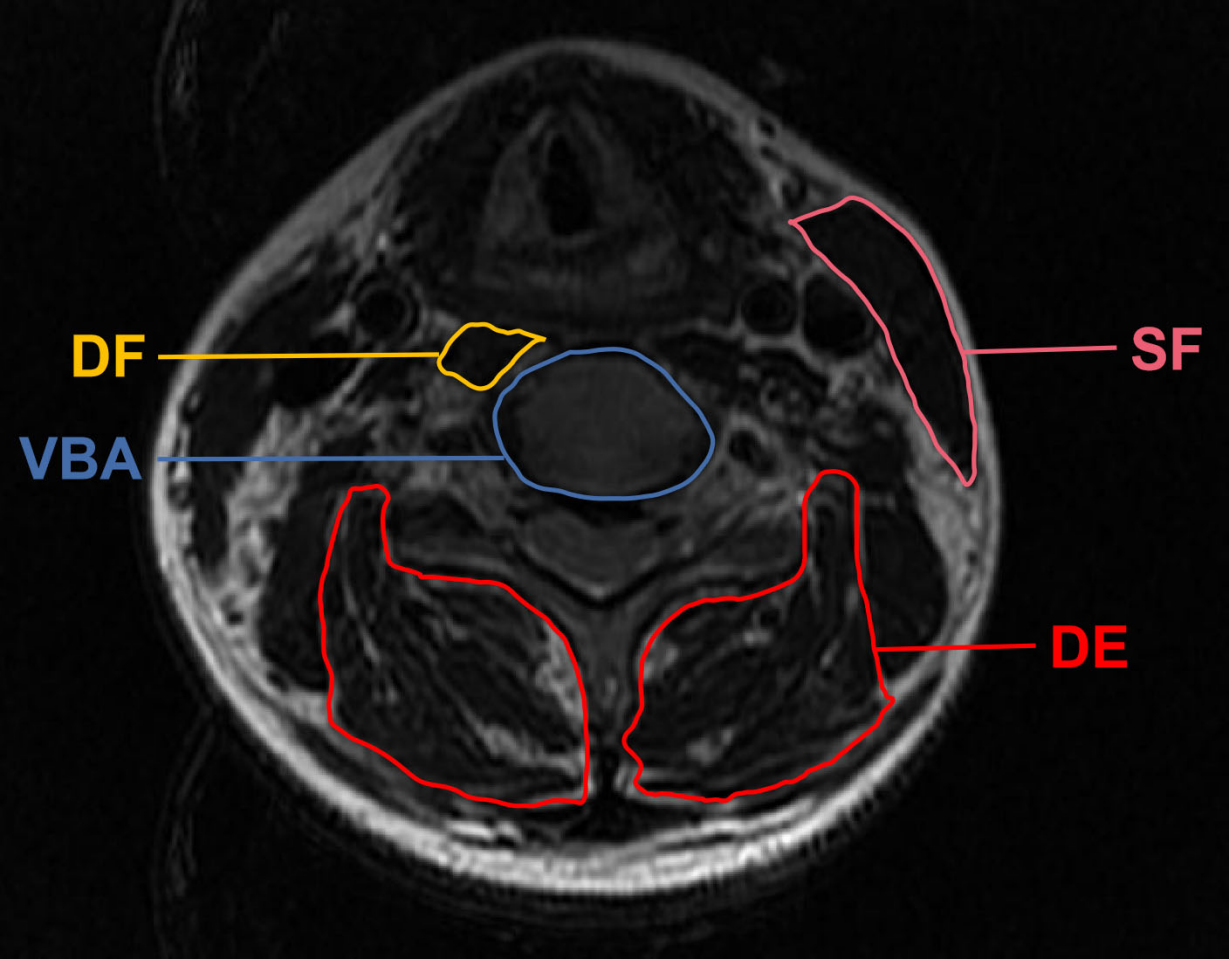
Demonstrative axial T2 magnetic resonance imaging sections of the regions of interest applied in measuring the cross-sectional area. SF, superficial flexor; DF, deep flexor; DE, deep extensor; VBA, vertebral body area.

In addition, CSA measurement method was based on a technique standardized by Takayama et al. (4). The measured flexors included sternocleidomastoid (superficial flexor, SF) and longus colli (deep flexor, DF). The extensors included multifidus and erector spinae (deep extensor, DE). CSA was defined by manually tracing the fascial boundary of CPM bilaterally. Moreover, vertebral body area (VBA) was introduced to eliminate biases arising out of variations in physiques (**Figure 3**) (17). The image-processing software platform (Image J, National Institutes of Health, Bethesda, MD) utilized enabled us to outline and analyze the regions of interest of CPM and VBA with a graphic cursor. The final FI classification and morphometry data were averaged between bilateral paraspinal muscles.

### Statistical analysis

Statistical analysis was performed using SPSS software version 24.0 (SPSS, Chicago, IL, USA). P < 0.05 was considered statistically significant. Continuous data are presented as mean ± standard deviation, while frequency data are expressed as counts or percentages. One-way analysis of variance (ANOVA) and Kruskal-Wallis H-test were utilized to compare continuous variables among groups. Correspondingly, *post-hoc* analysis was conducted using Tukey multiple or pairwise comparisons when the main effects were statistically significant. Frequency variables among groups were analyzed using chi-square and Fisher’s exact tests. Paired t-test and Wilcoxon signed-rank test were used for intra-group comparison at different time points. The 95% confidence interval (95% CI) is reported where applicable.

## Results

### Demographic and surgical data

The demographic and perioperative characteristics of the study cohort are presented in **Table 1**. The cohort consisted of 38 men and 72 women, with a mean age of 48.8 years (range, 29-67 years). The mean follow-up duration was 23.4 months (range, 12–93 months) and the mean operative time was 138.0 minutes (range, 90–300 minutes). A majority of the patients (76/110) had visible T2-weighted high signal intensity in the paracervical muscle at the evaluation level. A recent study demonstrated qualitative assessment of FI is preferable to quantitative CSA (16). Thus, for analysis the patients were categorized according to the fat infiltration: normal (Goutallier Grades 0-1), moderate (Goutallier Grade 1.5-2), and severe (Goutallier Grades 2.5-4). The most common FI grades were moderate and severe with 38 patients each, followed by normal with 34 patients. Notably, no significant difference was detected in age, sex, body mass index, alkaline phosphatase (ALP) level, involved level, mean length of follow-up, operation time, and blood loss among the groups (**Table 1**, P > 0.05).

### Clinical and radiographical outcomes

Regarding clinical outcomes, all three groups demonstrated significant improvement from pre-operation to the last follow-up (Paired t-test, P < 0.05), indicating that patients’ symptoms were relieved significantly after surgery. Nevertheless, ANOVA among groups revealed no statistically significant differences in terms of pre- and postoperative JOA, NDI, and VAS scores, as presented in **Table 2**.

**Table 2:** Clinical outcomes.

Variables	Normal (n=34)	Moderate (n=38)	Severe (n=38)	P Value
JOA scores				
Preoperative †	11.3 ± 1.4	10.9 ± 1.8	11.6 ± 1.6	0.237
Last follow-up †	16.1 ± 0.7‡	16.2 ± 0.7‡	16.2 ± 0.9‡	0.923
NDI scores				
Preoperative †	29.1 ± 4.4	30.2 ± 4.2	29.5 ± 3.6	0.545
Last follow-up †	8.0 ± 3.6‡	8.1 ± 3.4‡	7.0 ± 3.7‡	0.327
VAS scores				
Preoperative †	6.3 ± 1.5	6.6 ± 1.2	6.4 ± 1.3	0.509
Last follow-up †	1.2 ± 1.0‡	1.2 ± 1.0‡	1.2 ± 1.0‡	0.977

JOA, Japanese Orthopedic Association; NDI, Neck Disability Index; VAS, Visual analog scale.

† One-way analysis of variance.

‡ Paired t-test, compared with pre-operation, P<0.05.

Regarding radiographical outcomes, the three groups had similar overall and segmental mobility at all follow-ups. Globally, all groups experienced a significant decrease in ROM C2-C7 in the immediate postoperative period (paired t-test and Wilcoxon signed-rank test, P < 0.05), and ROM C2-C7 returned toward preoperative levels at the final follow-up. Likewise, paired-sample tests showed no statistically significant differences in the arthroplasty disc ROM between pre- and post-operation values (**Table 3**).

**Table 3:** Radiographical outcomes.

Variables	Normal (n=34)	Moderate (n=38)	Severe (n=38)	P Value
Cervical Sagittal Balance				
CL (°)				
Preoperative ‡	9.7 ± 9.5	5.2 ± 8.9	3.9 ± 10.1	**0.030**
Immediate Postoperative ‡	15.8 ± 9.1§	13.5 ± 7.6§	12.2 ± 8.2§	0.175
Last follow-up †	10.8 ± 7.4	8.6 ± 6.2§	7.0 ± 7.9§	0.079
SVA (cm)				
Preoperative ‡	1.5 ± 0.8	1.9 ± 0.9	1.9 ± 1.1	0.144
Immediate Postoperative ‡	2.1 ± 0.9§	2.2 ± 1.0	2.5 ± 1.2§	0.247
Last follow-up †	1.8 ± 0.7§	2.2 ± 1.0	2.3 ± 1.2§	**0.046**
TS (°)				
Preoperative ‡	21.5 ± 5.6	21.9 ± 4.9	20.2 ± 6.4	0.441
Immediate Postoperative ‡	25.2 ± 5.3§	25.2 ± 4.9§	24.3 ± 6.1§	0.708
Last follow-up ‡	22.7 ± 4.5	21.7 ± 5.5	21.7 ± 6.7	0.719
FSUA of Fusion Level (°)				
Preoperative ‡	-0.5 ± 5.5	-0.9 ± 4.3	-1.4 ± 5.6	0.740
Immediate Postoperative ‡	4.0 ± 5.8§	2.3 ± 4.9§	2.5 ± 5.0§	0.354
Last follow-up ‡	2.5 ± 5.7§	1.2 ± 3.8§	0.9 ± 5.5§	0.391
FSUA of Arthroplasty Level (°)				
Preoperative ‡	0.99 ± 4.2	-0.9 ± 5.6	-1.3 ± 5.1	0.124
Immediate Postoperative ‡	4.8 ± 4.7§	3.3 ± 5.3§	1.6 ± 4.8§	**0.022**
Last follow-up ‡	2.9 ± 4.2§	0.4 ± 6.0§	-1.9 ± 5.0	**0.001**
Arthroplasty Disc Angle (°)				
Preoperative †	3.4 ± 3.8	1.6 ± 3.9	1.1 ± 3.1	0.051
Immediate Postoperative †	6.4 ± 4.3§	5.6 ± 4.0§	3.8 ± 3.8§	**0.012**
Last follow-up †	3.5 ± 3.9	2.1 ± 3.7	0.9 ± 3.2	**0.015**
Cervical Sagittal Motion				
ROM C2-C7 (°)				
Preoperative †	49.9 ± 13.1	45.4 ± 14.9	48.0 ± 13.3	0.358
Immediate Postoperative †	26.1 ± 9.1§	23.7 ± 9.9§	27.5 ± 12.6§	0.165
Last follow-up ‡	43.7 ± 9.7§	42.9 ± 11.5	40.8 ± 9.8§	0.462
Arthroplasty Disc ROM (°)				
Preoperative †	9.7 ± 4.3	8.6 ± 3.9	9.1 ± 3.9	0.411
Immediate Postoperative †	6.3 ± 3.7§	6.2 ± 4.1§	5.7 ± 3.6§	0.762
Last follow-up ‡	8.1 ± 3.8§	9.1 ± 3.9	7.0 ± 3.7§	0.055

CL, cervical lordosis; SVA, sagittal vertical axis; TS, T1 slope; FSUA, function spine unit angle; ROM, range of motion.

† Kruskal-Wallis H-test.

‡ One-way analysis of variance.

§ Paired t-test and Wilcoxon signed-rank test, compared with pre-operation, P < 0.05.

Bold values denote statistical significance at the P < 0.05 level.

### Effect of FI on cervical sagittal balance

**Table 3** demonstrates cervical sagittal alignment parameters of different groups. CL was significantly higher in the normal group than in the severe cohort before surgery (multiple comparisons, p = 0.029, 95% CI: 0.49-11.18), and the difference was on the verge of significance between these two groups at the last follow-up (Mann-Whitney U test, p = 0.065). Notably, SVA did not indicate a significant difference between groups before or immediately following surgery but differed significantly afterward (Kruskal-Wallis H-test, p = 0.046). Furthermore, multiple comparisons revealed that FSUA of arthroplasty level was significantly lower in the severe group than in the normal group (immediate postoperative, p = 0.016, 95% CI: 0.50-6.05; last follow-up, p<0.001, 95% CI: 1.92-7.71). Similarly, follow-ups revealed statistically significant differences in ADA among various groups, particularly between normal and severe groups (immediate postoperative, p = 0.014; last follow-up, p = 0.012). However, no significant differences in FSUA of fusion level or TS were observed among the three groups at each follow-up period.

### Relationship between FI and CSA

The associations between FI level and CSA parameters are presented in **Table 4**. Collectively, there was no significant increase or decrease in DF and DE areas among different grades of FI classification (Kruskal-Wallis H-test, P > 0.05). Notably, a significantly higher mean SF area was identified in the normal group (3.7 ± 1.1 cm^2^) than in the severe FI group (3.0 ± 0.7 cm^2^) (pairwise comparisons, p=0.009). However, after VBA correction, SF ratio revealed no significant correlation with FI (Kruskal-Wallis H-test, P > 0.05). Meanwhile, neither SE nor DE ratios were associated with FI levels.

**Table 4:** Relationship between fatty infiltration and CSA.

Variables	Normal (n=34)	Moderate (n=38)	Severe (n=38)	P Value
CSA				
SF area (cm^2^) †	3.7 ± 1.1	3.4 ± 0.9	3.0 ± 0.7	**0.012**
DF area (cm^2^) †	0.4 ± 0.1	0.3 ± 0.1	0.3 ± 0.1	0.476
DE area (cm^2^) †	9.7 ± 2.7	9.6 ± 2.0	9.2 ± 2.2	0.567
CSA ratio				
SF area / VBA†	0.9 ± 0.2	0.9 ± 0.2	0.8 ± 0.2	0.116
DF area / VBA †	0.1 ± 0.0	0.1 ± 0.0	0.1 ± 0.0	0.693
DE area / VBA †	2.3 ± 0.5	2.5 ± 0.6	2.5 ± 0.3	0.121

CSA, cross-sectional area; SF, superficial flexor; DF, deep flexor; DE, deep extensor; VBA, vertebral body area.

† Kruskal-Wallis H-test. Bold values denote statistical significance at the P < 0.05 level.

## Discussion

This study is the first to investigate the influence of CPM in various statuses on clinical and radiographical outcomes following HS based on axial MRI. As stated in numerous studies, CPM degeneration is highly prevalent and has received much less attention than the lumbar spine (3, 4, 6, 9, 11, 15, 16). The results of our study reveal that fatty degeneration of the paraspinal musculature (Goutallier Grade ≥1.5) is up to 69.1%, of which 34.5% (Goutallier Grade ≥2.5) were relevant to the results. This finding is consistent with previously published data indicating that asymptomatic individuals might own fewer FI than CDDD patients (17, 24). Moreover, this study demonstrates that CSA of posterior extensor muscles (947.4 ± 230.5 mm^2^) was much lower in our cohort than in asymptomatic East Asians reported by Okada et al. at C5/6 level (1,599.6 ± 364.3 mm^2^) (3), implying an intimate relationship between CDDD and neck muscles (9, 11). Additionally, in agreement with studies examining paraspinal muscle (4, 11, 25), our findings reveal that FI is more prevalent in elderly patients. In contrast to earlier findings (26), however, no evidence of association between FI and gender or BMI was detected in our research.

In the current study, improved clinical outcomes reflected by VAS, JOA score, and NDI demonstrated that HS effectively ameliorated neurological symptoms, consistent with previous findings reported by Scott-Young et al. (27). More importantly, numerous studies in the past linked muscle FI to postoperative pain or neurological deficits, particularly following lumbar spine and posterior cervical surgery (4, 13, 26). In a recent systematic review of 873 patients, Jermy et al. recently identified a link between a low FI and greater improvement in low back pain and disability following lumbar spine surgery (12). In addition, De Pauw et al. revealed that chronic neck pain patients tend to develop muscle FI over time (8). However, these results are contradicted by the present study, which found that FI of CPM had no impact on patient-reported outcomes after HS. One possible explanation for our findings is that anterior surgery results in less obstruction to the paraspinal muscle (26). Additionally, compared with single-level CDDD, the clinical recovery of HS patients in the present study following multi-level decompression would theoretically be more significant. Similar to our findings, Pinter et al. reported that neither EQ-5D nor RAND scores distinguished between Goutalier grades in patients undergoing 1- to 3-level ACDF and postoperative NDI scores Goutalier 0-1 and Goutalier 2.5-4 were in close proximity (25.3 vs. 25.1). More surprisingly, their results indicated that patients with worse FI might benefit more from improved symptom relief (15).

**Figure 4:**
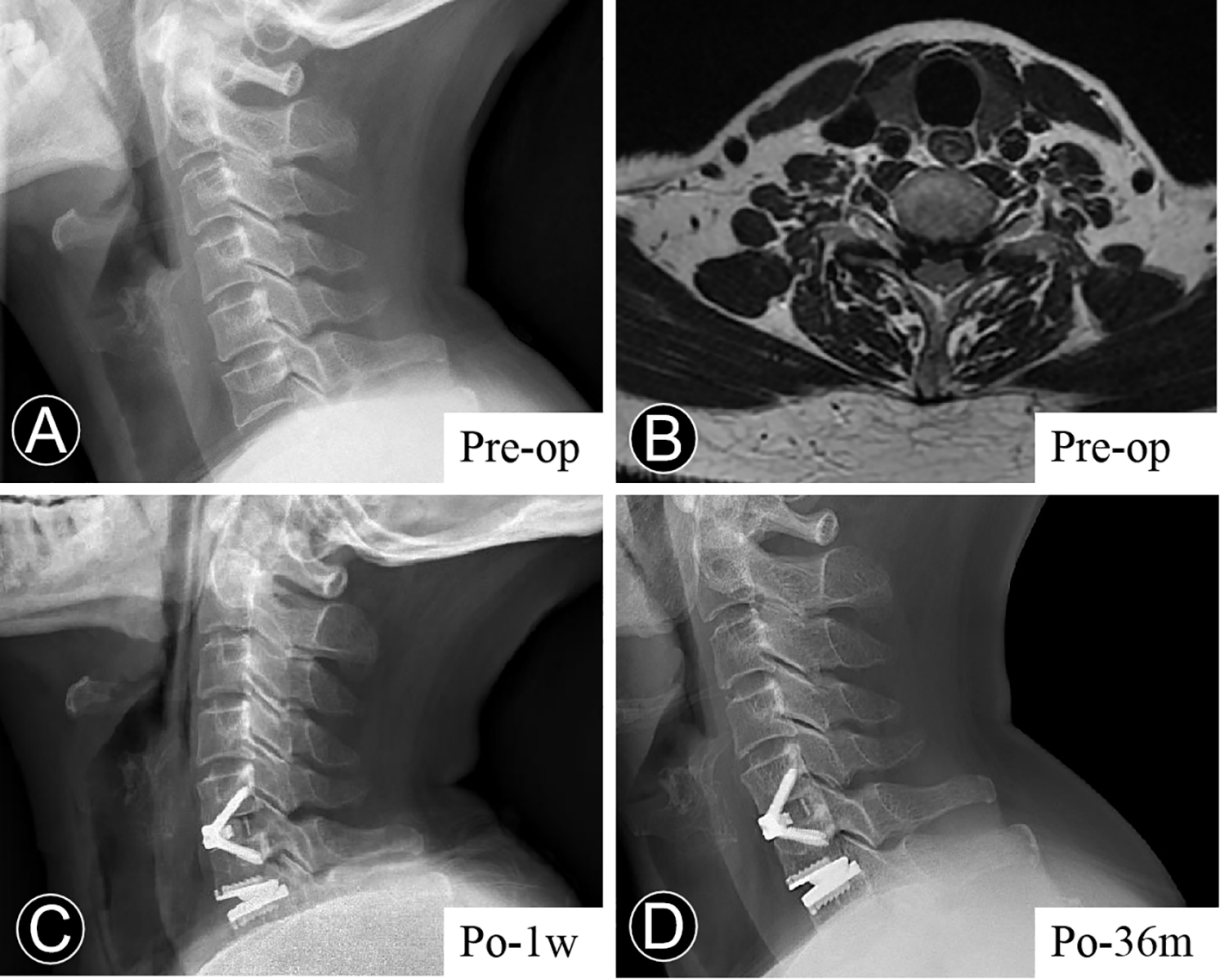
Representative case. Serial radiological examinations of a 67-year older woman with neck pain for more than two years. **(A)** Lateral view, showing that the preoperative cervical lordosis was 4.78°, and C2-7 sagittal vertical axis 4.01 cm. **(B)** T2-weighted axial MRI section, presenting a fatty infiltration degree of Goutalier 3-4. **(C)** The immediate postoperative lateral radiograph demonstrated a relatively satisfactory sagittal alignment. **(D)** At the last follow-up, the lateral radiograph demonstrated the loss of cervical lordosis and an increase in the sagittal vertical axis.

An additional finding of our study is the role of CPM in cervical sagittal alignment. Mitsutake et al. demonstrated that fat within cervical multifidus muscle could directly cause postural instability in static standing (28). Similarly, Tamai et al. conducted a propensity score-matched analysis of 1500 patients and revealed that severe muscle FI at C7 was an independent characteristic of patients with cervical imbalance (SVA≥40 mm) (29). In continuation of their research background, Tamai and his colleagues also found FI ratio of CPM at the mid and lower cervical level significantly correlated with cervical balance parameters (neck tilt and thoracic inlet angle) (11). Moreover, a multivariate regression analysis by Passias et al. (30) determined that preoperative FI of CPM was a strong predictor of postoperative sagittal cervical imbalance, specifically for SVA. However, the muscle-related difference in cervical sagittal alignment remains controversial. A previous cross-sectional study demonstrated no association between cervical lordotic alignment and muscle fatty degeneration (31). Moreover, our findings are similar to those presented by Inoue et al. (31), who observed that fatty degeneration had almost no impact on cervical or segmental movement. Nevertheless, our results indicated that severe FI in the cervical extensor muscles was significantly associated with higher SVA at the final follow-up. Additionally, the severe FI group had a trend toward lower CL pre- and postoperatively. Evidence from the lumbar spine has also established the link between lumbar lordosis and FI (4, 32). A biomechanical study by Patwardhan et al. (33) investigated kinematic, kinetic, and muscular responses to cervical sagittal imbalance, presenting increased SVA to be associated with shortening of the cervical flexor and lengthening of the cervical extensor. Notably, when considering the arthroplasty level, segmental alignment was linked to FI, implying that patients with severe FI may be unsuitable for CDA (**Figure 4**), as FSUA of fusion level was maintained well in all groups during follow-up. This is probably because annulus fibrosus of intervertebral disc experiences multidirectional tension *in vivo*, yet the ball-joint artificial prosthesis like Prestige-LP lacks physiological tensile properties (34).

The association between FI and CSA remains contentious. Based on measurements of paraspinal muscle morphology of 160 subjects, Takayama et al. (4) revealed a negative correlation between CSA and FI. Likewise, the present study identified a difference in SF area among the three groups. However, in contrast to Pinter et al. (16), our finding presented that DF and DE areas were similar between normal and severe groups. Moreover, after adjusting for VBA, all CPM areas did not differ significantly between the groups. Notably, previous research revealed that worsening CSA ratio is significantly correlated with greater segmental kyphotic change (17). Consequently, CPM investigation cannot be limited to fat infiltration, and further studies focusing on CSA of CPM are required to analyze its relevance with outcomes following HS or CDA. With the continuous progression of artificial intelligence, quantification of MRI based metric of muscle composition will contribute to analyses of CSA and FI in all paraspinal muscles (35).

This study has several limitations. First, this study demonstrated no direct evidence between FI and muscle strength decrease. Additionally, our analysis found that BMI was unrelated to FI. Therefore, muscle strength measurements are required in the future for studying neck muscles. Moreover, both the anterior and posterior paravertebral muscles are reported to contribute to the maintenance of cervical posture and control intervertebral motion (26). In addition to assessing ratio of CSA, more quantitative and qualitative indicators are warranted. Furthermore, neck muscle exercises have been revealed to be effective in improving postoperative outcomes of patients (36), but as a retrospective study, this study lacks corresponding intervention methods.

## Conclusion

This study demonstrated that CPM degeneration is common and age-related in CDDD patients. More importantly, severe FI of CPM is linked to postoperative sagittal balance disorder in HS patients, particularly at the arthroplasty level. However, as evaluated by FI grade, the alterations in CPM appear to have no impact on clinical outcomes and reveal small correlations to CSA.

## Data availability statement

Summarized data have been presented in this manuscript. The raw data for this study are located and protected at West China Hospital of Sichuan University. Sharing of the raw data is not suggested, because a secondary analysis is planned.

## Ethics statement

The studies involving human participants were reviewed and approved by Biomedical Research Ethics Committee, West China Hospital of Sichuan University. The patients/participants provided their written informed consent to participate in this study.

## Author contributions

JH and TW provided equal contributions to this study, both analyzed and interpreted all data, performed statistical analysis and prepared the manuscript. Analysis of radiographs was performed by BW and YH. CD helped in the statistical analyses. HL designed and supervised the study and was a major contributor to the preparation of the manuscript. All authors have read and approved the final manuscript.
